# Nitroglycerin application and coronary arteriogenesis

**DOI:** 10.1371/journal.pone.0201597

**Published:** 2018-08-17

**Authors:** Nora Gatzke, Philipp Hillmeister, André Dülsner, Nadija Güc, Rica Dawid, Katherine H. Smith, Nikolaos Pagonas, Peter Bramlage, Michaela Gorath, Ivo R. Buschmann

**Affiliations:** 1 Department for Angiology, Brandenburg Medical School, Campus Brandenburg/Havel, Brandenburg/Havel, Germany; 2 Department of Cardiology, Charité University Hospital, Campus Virchow, Berlin, Germany; 3 Center for Cardiovascular Research (CCR) Charité University Hospital, Campus Mitte, Berlin, Germany; 4 Institute for Pharmacology and Preventive Medicine, Mahlow, Germany; 5 G. Pohl-Boskamp GmbH & Co. KG, Hohenlockstedt, Germany; Centro Cardiologico Monzino, ITALY

## Abstract

**Background:**

In the presence of a coronary occlusion, pre-existing small collateral vessels (arterioles) develop into much larger arteries (biological bypasses) that have the potential to allow a certain level of perfusion distal to the blockage. Termed arteriogenesis, this phenomenon proceeds via a complex combination of events, with nitric oxide (NO) playing an essential role. The aim of this study was to investigate the effects of supplemental administration of NO donors, i.e., short-acting nitroglycerin (NTG) or slow-release pelleted isosorbide dinitrate (ISDN), on collateral development in a repetitive coronary artery occlusion model in rats.

**Methods:**

Coronary collateral growth was induced via a repetitive occlusion protocol (ROP) of the left anterior descending coronary artery (LAD) in rats. The primary endpoints were the histological evaluation of rat heart infarct size and ST-segment elevation (ECG-analysis) upon final permanent occlusion of the LAD (experimentally induced myocardial infarction). The effects of NTG or ISDN were also evaluated by administration during 5 days of ROP. We additionally investigated whether concomitant application of NTG can compensate for the anti-arteriogenic effect of acetylsalicylic acid (ASA).

**Results:**

After 5 days of ROP, the mean infarct size and degree of ST-elevation were only slightly lower than those of the SHAM group; however, after 10 days of the protocol, the ROP group displayed significantly less severe infarct damage, indicating enhanced arteriogenesis. Intermittent NTG application greatly decreased the ST-elevation and infarct size. The ISDN also had a positive effect on arteriogenesis, but not to the same extent as the NTG. Administration of ASA increased the infarct severity; however, concomitant dosing with NTG somewhat attenuated this effect.

**Conclusion:**

Intermittent treatment with the short-acting NTG decreased the size of an experimentally induced myocardial infarct by promoting coronary collateral development. These new insights are of great relevance for future clinical strategies for the treatment of occlusive vascular diseases.

## Introduction

It is many years since the first definitive evidence of the presence of coronary collateral vessels in humans was published [1]. Although found in both healthy and diseased hearts, the presence of a coronary occlusion results in larger diameter collateral vessels, with multiple layers of smooth muscle cells (SMCs) within an elastin-containing extracellular matrix (ECM) [2]. The ability of these collateral vessels to maintain a supply of blood distal to the coronary occlusion can result in a decrease in infarct size as well as reduced mortality [3, 4].

The remodelling of pre-existing small arterioles into larger collateral arteries (biological bypasses), a process termed arteriogenesis, has been attributed to the elevated blood flow resulting from the blockage in the coronary artery [5]. The resulting increase in shear stress activates the intimal endothelial cells in the collateral vessel, which produce nitric oxide (NO) as well as a variety of growth factors and cytokines. This leads to the recruitment of mononuclear cells, which form an inflammatory pro-arteriogenic environment that results in increased NO secretion, SMC proliferation and ECM production and remodelling [6–8], with NO playing a crucial role: Eitenmüller et al. demonstrated that inhibition of endogenous NO synthesis with N(G)-nitro-L-arginine methyl ester (L-NAME) abrogated shear-stress induced arteriogenesis [9]. Troidl et al. demonstrated the importance of two specific isoforms of NO synthase (NOS) by showing that the combined deletion of endothelial NOS (eNOS) and inducible NOS (iNOS) in mice completely blocked arteriogenesis [8]. A number of approaches to therapeutically enhancing arteriogenesis have been investigated. Application of angiogenic growth factors such as vascular endothelial growth factor (VEGF) gave disappointing results [7], while granulocyte-colony stimulating factor (G-CSF) and granulocyte-macrophage colony-stimulating factor (GM-CSF) have shown some promise [10, 11]. External counterpulsation (ECP) was developed in order to artificially enhance shear stress, with early data appearing encouraging [12]. Furthermore a previous study demonstrated increased arteriogenesis on continuous administration of a long-acting NO donor [8]. The results of these studies provided evidence that shear stress and particularly shear stress-triggered NO production appear to be the predominant physiological triggers for arteriogenesis.

Investigation of potential therapeutic approaches to increasing coronary collateralisation requires a suitable model. Repetitive application of ischaemia via temporary occlusion of a coronary artery has been shown to induce collateral development in rats, with this model used to evaluate the effect of G-CSF [10]. In the present study, we employed a similar repetitive occlusion protocol (ROP) in order to evaluate the effects of intermittent NTG and slow-release ISDN administration on the development of coronary collateral vessels and the extent of myocardial infarction on the application of a full permanent occlusion (FPO).

## Methods

### Study design

This was a randomised, blinded, placebo-controlled study in a non-myocardial-infarct arteriogenesis rat model. All investigators that administered the test compounds remained blinded to the group assignment throughout the test period, as did those performing the ECG and micro-CT analysis.

### Animal ethical approval details

Animal study permission was given by the Landesamt für Gesundheit und Soziales, Berlin, Germany (No. G0255/11 and G0013/13). The current study was performed in male Sprague—Dawley rats (300 to 350 g, Harlan—Winkelmann, Borchen, Germany) in conformity with the German Law for the Protection of Animals and the National Institute of Health Guidelines for Care and Use of Laboratory Animals.

### Animal preparation and implantation of coronary occlusion device

Male Sprague Dawley rats of approximately 300–350 g in weight were used for all experiments. Prior to surgery, the animals were sedated with ketamine (100 mg/kg [50 mg/ml]; i.p.) and xylazine (3 mg/kg [4 mg/ml]; i.p.) and then intubated. General anaesthesia was induced and maintained with isoflurane inhalation (1–2% in 100% oxygen). The animal was placed on its dorsal side and cutaneous clips were attached. These were linked to a BioAmp differential amplifier coupled to a PowerLab data acquisition system (AD Systems). ECG readings were monitored and recorded during the subsequent surgery.

The animal was placed on its right side and the heart was exposed using a left thoracotomy. A mini pneumatic snare occluder was implanted around the left anterior descending (LAD) coronary artery of each rat [13]. This device includes a mini latex balloon mounted within an umbrella sheath. The balloon is attached to a catheter to allow inflation and deflation from outside the cage of the animal. A prolene suture (5–0) is placed around the LAD and the deflated balloon to act as a snare. On inflation of the balloon (0.2 to 0.25 ml air), the suture is pulled up, compressing the artery against the balloon and stopping perfusion of the artery.

After implantation, correct function of the occluder was determined by direct observation of the left ventricle to check for blanching and hypokinesis, and by identification of ST-segment elevation on the ECG readings. The chest was then closed under positive end-expiratory pressure and residual air was evacuated from the thoracic cavity. The catheter of the occluder was externalised between the scapulae and protected and held in place with a stainless steel coil attached to a ring. Analgesia (buprenorphine 0.05 mg/kg; s.c.) and an antibiotic (enrofloxacin 10 mg/kg; s.c.) were administered and the animal was observed in a recovery cage for 2 h. It was then transferred to the animal care facility, where it was continuously monitored by technicians. Pain relief was provided by twice-daily administration of buprenorphine (0.5 mg/kg; s.c.) for the duration of the study.

### Repetitive occlusion protocol

The ROP commenced three days after implantation of the occluder device. The procedure was automated to provide 40 s of occlusion every 20 min for a period of 2 h 20 min. This was followed by a rest period of 5 h 40 mins, during which the balloon remained deflated. This sequence was repeated three times a day for 5 or 10 days. For the rats in the SHAM groups, the balloon was implanted but not inflated.

### Administration of test compounds

Nitroglycerin (NTG; glyceryl trinitrate) in an immediate release formulation (solution containing 8.3 mg NTG/g; G. Pohl-Boskamp GmbH & Co. KG, Hohenlockstedt, Germany) was used as an intermittently administered short-acting source of NO, with the carrier solution used as a placebo (NTG-PLACEBO). Isosorbide dinitrate (ISDN) in a slow-release formulation (retard pellets containing 200 mg ISDN/g; G. Pohl-Boskamp GmbH & Co. KG, Hohenlockstedt, Germany) was used as a long-acting source of NO, with neutral pellets used as a placebo (ISDN-PLACEBO). Acetylsalicylic acid (ASA) was purchased from Merck Chemicals (Germany) and dissolved in water for administration.

NTG and NTG-PLACEBO were administered oromucosally via the buccal route in order to avoid placing further stress on the rats. The assigned agent was administered immediately prior to the first two occlusions of each day, with the exception of the dosing regimen study, where it was administered prior to the first, first two, or all three occlusions. A dose of 17.37 μg was given per application (equivalent to 0.8 mg for a human), with alternative doses equivalent to 0.2 mg and 0.4 mg investigated in the dosing regimen study. The ISDN and ISDN-PLACEBO pellets were suspended in water for administration via peritoneal gavage. A dose of 2.6 mg (equivalent to 2 mg/kg body weight for a human) was given each morning immediately prior to the first occlusion. ASA was dissolved in water for administration via peritoneal gavage. An ASA dose of 2.22 mg was given each morning (equivalent to 100 mg/day for a human).

### ECG measurements

During the first occlusion at the beginning of the study, ECG readings were recorded for each rat. At the end of the study (5 or 10 days later), the animal was anaesthetised and an FPO was produced by maintaining balloon inflation for 90 min. ECG readings were recorded and the degree of ST-segment elevation and presence of any ventricular arrhythmias were documented. These arrhythmias were classified according to the methods of Lown [14], with the grade increasing with increasing severity.

### Triphenyltetrazolium chloride staining for infarct visualisation

After the final ECG measurements were taken, the chest of the anaesthetised rat was opened via mid-thoracotomy and the heart was excised. The tissue was sectioned from apex to base, parallel to the atrioventricular groove, producing 2-mm-thick slices. These were then incubated at 37°C for 20 min in sodium phosphate buffer (0.09 mol/l) containing 1% triphenyltetrazolium chloride (TTC) and 8% dextran. The slices were then fixed in 10% formaldehyde and imaged using a digital camera mounted on a stereomicroscope. The infarcted area, which appeared white, was quantified using a planimetric program (Adobe Photoshop), and expressed as a percentage of the area of the left ventricle.

### Statistical analysis

Data are given as means with standard deviations (SD). Between-group differences were analysed by ANOVA using SPSS 20 (IBM, NY, USA), with false discovery rate correction. A p-value of <0.05 was considered to be statistically significant.

## Results

### Effect of the repetitive occlusion protocol on ST-segment elevation and infarct size

**Fig 1:**
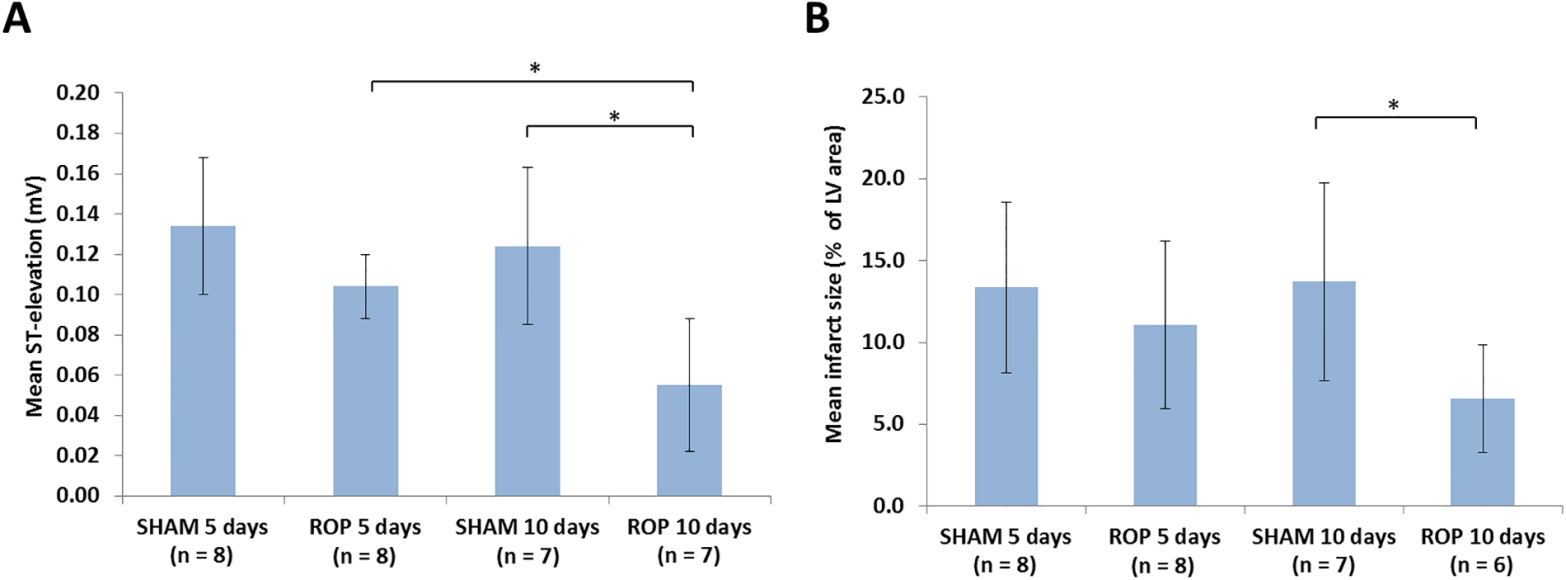
Effect of ROP on ST-elevation and infarct size upon full permanent occlusion. A) mean ST-segment elevation calculated from ECG readings taken during the formation of a full permanent occlusion (FPO) after 5 or 10 days of either SHAM or the repetitive occlusion protocol (ROP); B) mean infarct size resulting from the formation of an FPO after 5 or 10 days of either SHAM or the ROP. Error bars represent the standard error of the mean; *p-value of <0.05.

After 5 days, the mean ST-segment elevation calculated from the ECG readings taken during formation of the FPO was slightly lower for the animals that had received the ROP than those that had received the SHAM (Fig 1A). However, by 10 days, the ROP resulted in a significantly lower value (0.055 ± 0.033 vs. 0.124 ± 0.039 mV for the SHAM group; p < 0.05). Furthermore, ST-elevation was significantly lower after 10 days of the ROP than it was after only 5 days (0.055 ± 0.033 vs. 0.104 ± 0.016 mV; p < 0.05).

The mean size of infarcts present after the FPO that was carried out at 5 days did not differ greatly between the ROP and SHAM groups (Fig 1B). At 10 days, however, the ROP group had a significantly lower infarct area (6.57 ± 3.26% vs. 13.71 ± 6.04%; p < 0.05).

### Effects of intermittent NTG and sustained ISDN on ST-elevation, infarct size and ventricular arrhythmia

**Fig 2:**
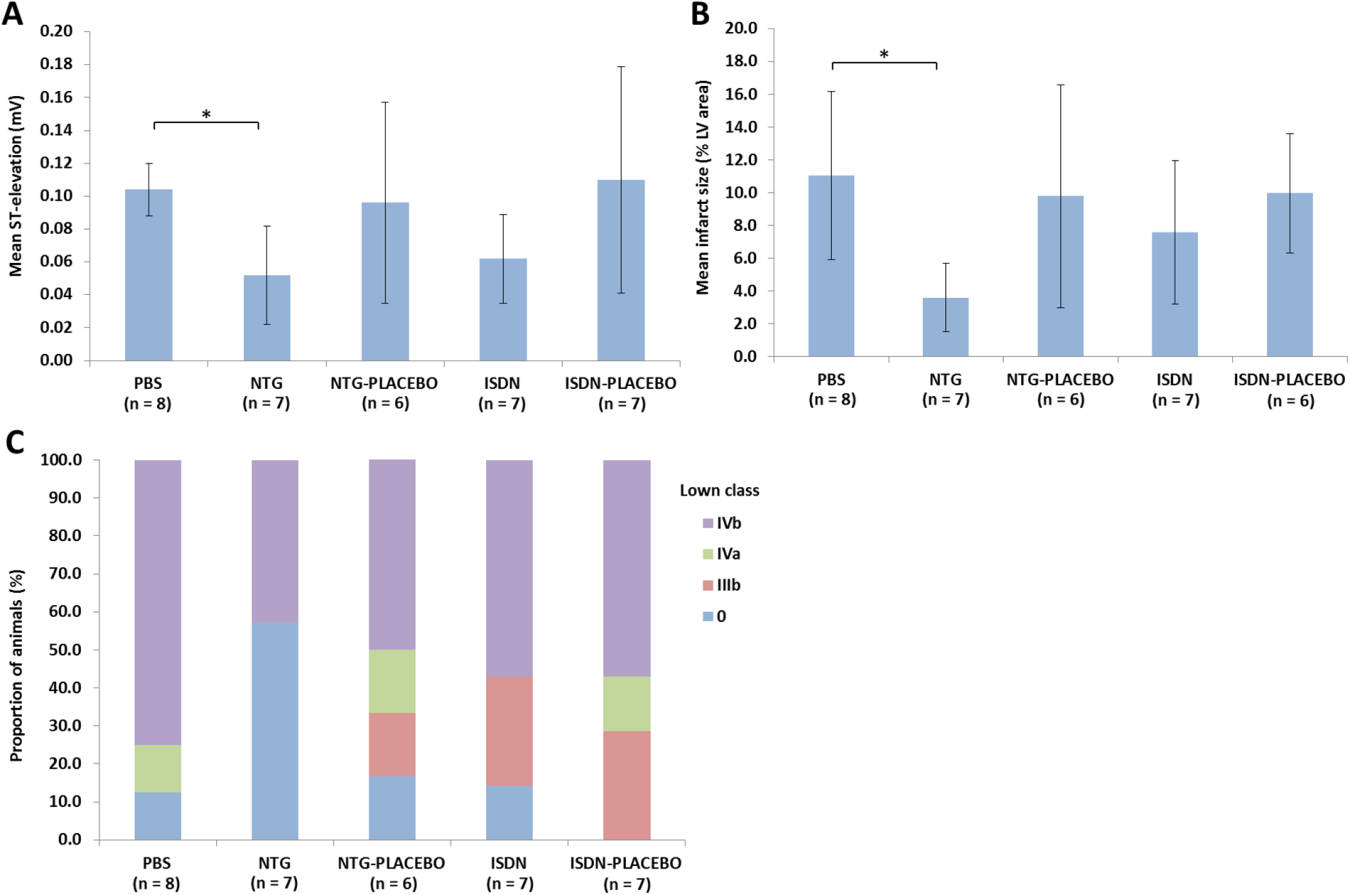
Effect of NTG and ISDN on ST-elevation, infarct size and ventricular arrhythmias (Lown class) upon full permanent occlusion after 5 days of ROP. A) mean ST-segment elevation; B) mean infarct size; C) class of ventricular arrhythmia (Lown [14]) on formation of a full permanent occlusion (FPO) after 5 days of the repetitive occlusion protocol (ROP) with administration of phosphate buffered saline (PBS) control, nitroglycerin (NTG; 0.8 mg human equivalent), isosorbide dinitrate (ISDN; 2 mg/kg human equivalent) or corresponding placebo. *p-value of <0.05.

Compared to the PBS control, intermittent administration of short-acting NTG resulted in a significantly lower ST-elevation in the ECG readings taken during formation of an FPO at 5 days (0.104 ± 0.016 vs. 0.052 ± 0.030 mV; p < 0.05; Fig 2A). When slow-release ISDN was applied, the ST-elevation (0.062 ± 0.027 mV) was again lower than with the PBS control, but not to a statistically significant extent. Infarct size followed the same trend, with values of 11.05 ± 5.12% and 3.61 ± 2.08% calculated for the PBS and NTG groups, respectively (p < 0.05), and 7.59 ± 4.38% for the ISDN group (Fig 2B).

The majority of the animals that received the PBS control during 5 days of the ROP displayed ventricular arrhythmias at Lown class IVb during the FPO (Fig 2C). The only group in which a significant proportion of animals displayed no arrhythmias was the NTG group.

### Effect of NTG dosing regimen on ST-elevation

**Fig 3:**
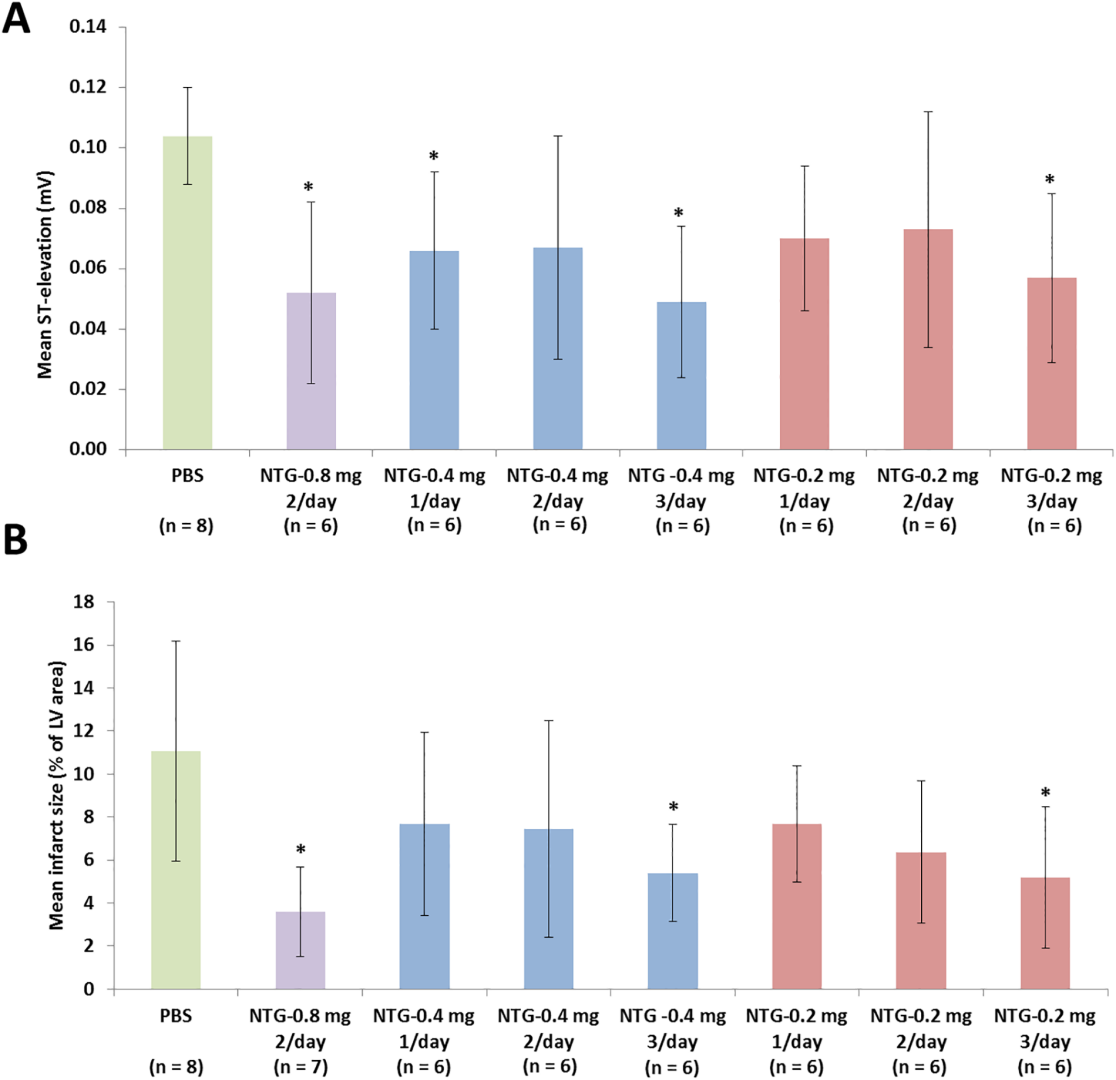
Effect of NTG dosing regimen on infarct size and ST elevation upon full permanent occlusion after 5 days of ROP. A) mean ST-segment elevation and B) mean infarct size upon formation of a full permanent occlusion (FPO) after 5 days of the ROP with administration of different dosages (human equivalents) of nitroglycerin (NTG) before the first, first two, or all three sets of occlusions of each day. *p < 0.05 compared to PBS control.

After 5 days of different NTG dosing regimens, all groups of animals displayed lower ST-elevation during FPO than the animals that received the PBS control (Fig 3A). The difference was most notable for the regimens that included three doses per day, with the 0.4 mg (0.049 ± 0.025 mV) and 0.2 mg (0.057 ± 0.028 mV) doses resulting in significantly less elevation compared to the PBS (0.104 ± 0.016 mV). The mean infarct size also varied with the number of applications per day, with the three-per-day groups showing significantly smaller infarcted areas than the PBS control (Fig 3B). There appeared to be a non-significant trend to a lower infarct size with increasing number of applications, which did not differ between the 0.2 mg and 0.4 mg dosages.

**Fig 4:**
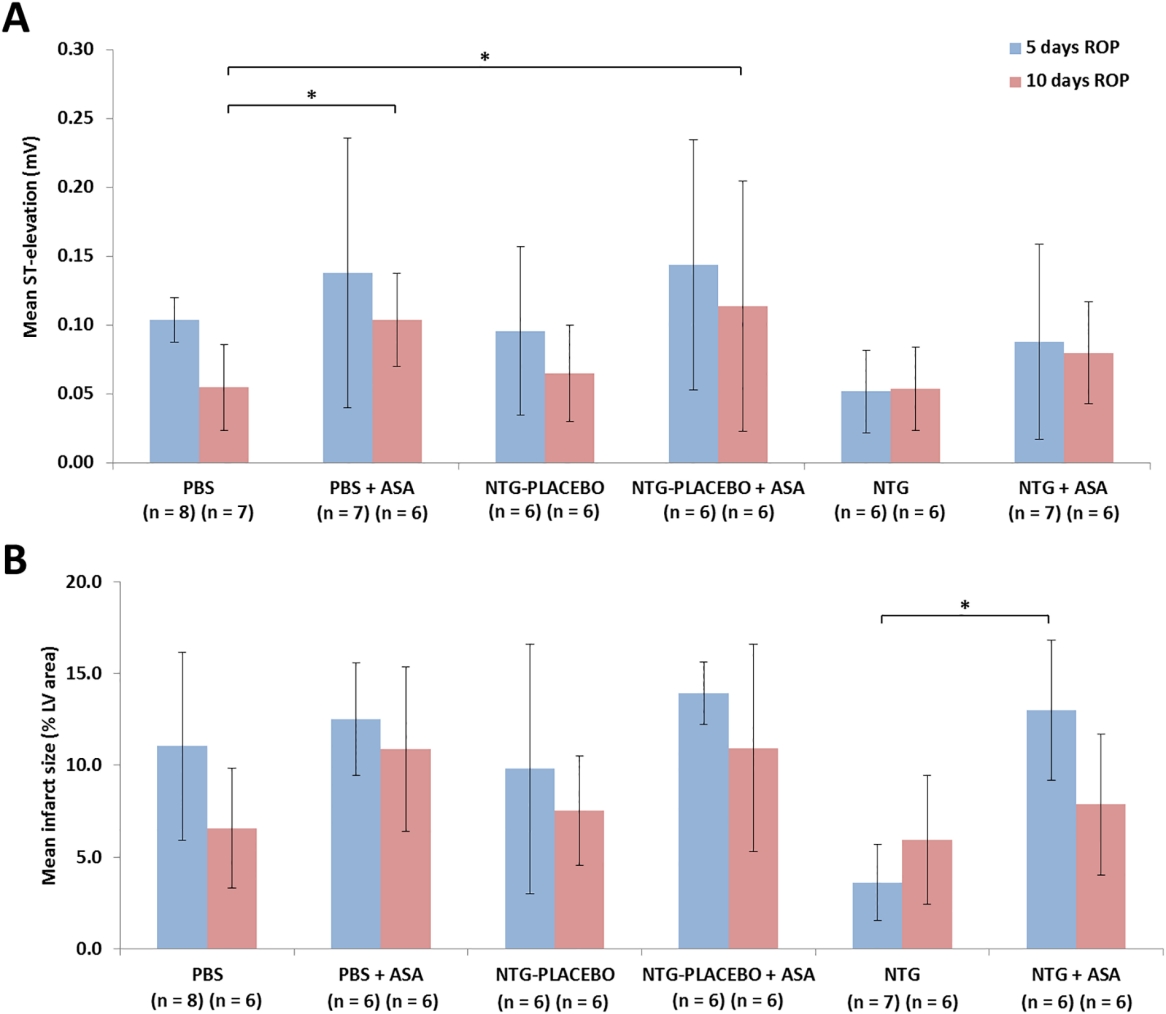
Effect of ASA and NTG on ST-elevation and infarct size upon full permanent occlusion. A) mean ST-segment elevation; B) mean infarct size upon formation of a full permanent occlusion (FPO) after 5 or 10 days of the ROP with administration of phosphate buffered saline (PBS) control, nitroglycerin (NTG; 0.8 mg human equivalent), or placebo to NTG, with and without co-administration of acetylsalicylic acid (ASA; 100 mg human equivalent).

After 5 and 10 days of the ROP, the mean ST-elevations for the PBS, NTG-PLACEBO and NTG groups were slightly lower than for the corresponding groups at the same time point that were additionally administered ASA (Fig 4A). The difference was particularly notable at 10 days, where the addition of ASA to the PBS control produced a significantly higher ST-elevation (0.055 ± 0.031 vs. 0.104 ± 0.034 mV; p < 0.05). At 5 days, the size of infarct after FPO did not vary significantly between the PBS and NTG-PLACEBO groups with and without ASA (Fig 4B). However, the administration of ASA with NTG greatly increased the infarct size compared to the use of NTG alone (13.00 ± 3.82% vs. 3.61 ± 2.08%; p < 0.05). After 10 days of the ROP, the infarct size for the group administered NTG in combination with ASA (7.86 ± 3.85%) appeared smaller than when the ASA was given alone (10.89 ± 4.48%) or with NTG-PLACEBO (10.93 ± 5.64%), although the differences did not achieve statistical significance.

## Discussion

The ROP protocol resulted in reduced severity of infarct compared to the SHAM procedure, indicating increased collateral blood flow. Administration of short-acting intermittent NTG and slow-release ISDN appeared to increase the vessel diameter further, with this being more significant for the former. When the animals were given ASA, infarct size was greater than for the PBS and placebo controls; however, co-administration of NTG attenuated this to a certain extent.

Application of the ROP resulted in a lowering of ST-elevation and a decrease in infarct size when an FPO was generated. This was evident after 5 days of the protocol, and became significant after 10 days. This indicates a gradual increase in the size of the pre-existing collateral vessels and has been previously demonstrated in a similar repetitive ischaemia model in rats [13]. In this prior study, Toyota et al. used micro-CT imaging to demonstrate the maturation of coronary collaterals, and reported increased blood flow after 10 days of the repetitive ischaemia protocol. Initiation of the process of collateral remodelling has been attributed to elevated shear stress applied to the vessel walls [5], such as that present during the 40 seconds of each temporary occlusion during the ROP in the present study. Elevated shear stress is known to activate the endothelial lining, resulting in the many processes that contribute to arteriogenesis [5, 7]. A major process involves the production of NO, which on the one hand dilates the vessels and on the other hand encourages collateral maturation. In accordance with this, inhibition of endothelial NO synthase has been demonstrated to reduce the NO-mediated acute dilation of peripheral collateral vessels occurring in response to increased blood flow produced by an occlusion as well as to abrogate shear-stress induced arteriogenesis [9, 15].

Because of the strong inhibitory effects of NO synthase inhibitors on arteriogenesis and the results of a previous study of Troidl et al., which demonstrated greatly increased collateral blood flow after formation of a femoral artery ligation and continuous infusion of the long-acting NO donor DETA NONOate in a rabbit model [8], we intended to investigate the effect of supplementary administration of exogenous NO on collateral development. In particular we wanted to pursue the question, if an intermittent short-term administration of exogenous NO is also promoting arteriogenesis. Therefore we were challenged to investigate the arteriogenic effect of a short-acting NO donor in comparison to a long-acting NO donor.

We chose two clinically well-established organic nitrates as short- and long-acting sources of NO, i.e. immediate-release, transmucosal NTG and slow-release, oral ISDN and applied them prior to the first two (NTG) or first (ISDN) occlusions of the ROP each day. Thereby NTG was expected to mimic the short-term shear stress-mediated release of endogenous NO during physical activity and was administered to achieve NO release during ROP, while ISDN aimed at a long-term NO elevation lasting markedly longer than the ROP. The effects of these treatments were evaluated after 5 days of ROP, as at this time point the decrease in infarct severity was not statistically different between the ROP and SHAM control groups, which allowed for a more accurate assessment of the effects of either drug.

Surprisingly, we found that short-acting NTG showed a more pronounced effects compared to ISDN with regard to the study endpoints. Compared to the PBS control, NTG administration resulted in a significantly reduced severity of the infarction as well as in a significantly lower ST-elevation induced by the FPO after the 5 days, indicating enhancement of arteriogenesis. Furthermore, the proportion of animals displaying no ventricular arrhythmias was much higher for the rats that received the short-acting NTG than for those that received PBS or the placebo. We also found that slow-release ISDN reduced ST-elevation during the infarct initiated after 5 days of ROP, which is in agreement with the previous study by Troidl et al. [8]. In the present work, the size of the infarct in the animals that received ISDN was not significantly smaller than in the placebo or PBS controls. Furthermore, the proportion of animals that displayed no arrhythmias was similar to the PBS control group. This suggests that NTG stimulated arteriogenesis to a greater extent than did the slow-release ISDN. These findings are of high relevance as they demonstrate for the first time a pro-arteriogenic effect by the intermittent administration of a short-acting NO donor, i.e. NTG, an important feature separating our study from previous reports. Furthermore we could demonstrate for the first time that arteriogenesis can be triggered by the administration of organic nitrates, well-known drugs in the symptomatic pharmacotherapy of coronary artery disease. In contrast to the present study earlier attempts to stimulate arteriogenesis by NTG administration failed, probably because of the induction of tolerance development due to continuous infusion of NTG [8].

The distinct effects of NTG and ISDN on arteriogenesis in our study suggest an influence of the temporal kinetic of supplemental NO delivery on the degree of the arteriogenic effect, e.g. regarding the point of time of administration as well as regarding the duration of NO release by the NO donor. A possible explanation for the distinct effects in our study setting is that administering the NTG bolus shortly before the first two ROP-occlusions of each day would expose the blood vessel walls to a high concentration of NTG or NO respectively simultaneous to the increased shear stress caused by the applied occlusions. This combination of both stimuli may result in a greater pro-arteriogenic effect than that due to the ISDN, which would release NO for a prolonged period of time. A pulsatile, burst-like release of NO might have a greater impact on arteriogenesis than a long-term, continuous NO delivery. In accordance to this the administration of the short-acting NO donor NTG in this setting may mimic the physiological processes during physical strain, i.e. the endogenous release of NO upon physical training, of an occlusive artery disease patient more accurately.

Because both NO donor drugs NTG and ISDN are potent vasodilators, vasodilation may play a specific role regarding the observed pro-arteriogenic effect of NTG and ISDN. The combination of exogenous NO delivery by NTG administration and simultaneous endogenous shear stress-mediated NO production during the occlusion may result in greater vasodilation than that due to long-term NO release of slow-release ISDN, which would persist also independent of ROP-occlusion. This theory could be also corroborated by the greater decrease in infarct severity shown for the animals who received 0.8 mg (human equivalent) of NTG twice a day compared to 0.4 or 0.2 mg (human equivalent) twice a day. However, independent from the mechanism, this sheds a light on the influence of the dosage of the administered NO donor regarding the promotion of arteriogenesis since it appears that the lower doses did not provide as strong a stimulus as the 0.8 mg dose.

Vasodilation is triggered by the activation of soluble guanylyl cyclase (sGC) by NO, leading to accumulation of cyclic guanosine-3’,-5’-monophosphate (cGMP), with subsequent downstream signalling ultimately resulting in SMC relaxation [16]. However, there has been some suggestion that NTG may cause vasodilation via an NO-independent mechanism [17, 18], with alternative species derived from the nitrate being responsible for activation of sGC [16]. These doubts arose due to the circumstance, that efforts to detect NTG-derived NO in blood vessels failed in the past [17, 19, 20], whereas NO from donors such as DETA-NONOate was more freely available for detection as it is produced by spontaneous dissociation at physiological pH [8].

However, Opelt et al. recently provided further evidence that NTG-derived NO fully accounts for the activation of vascular sGC by NTG [21]. They suggested that the lack of NO detection in other studies may have been due to the cytosolic co-localisation of sGC with aldehyde dehydrogenase (ALDH)-2, which is involved in the biotransformation of NTG.

Hence the mechanism of action of endogenous NO in the stimulation of arteriogenesis is still unclear, also other effects could be causative. It is therefore questionable which NO mediated action/actions may be causal for the pro-arteriogenic effect of the two organic nitrates and also why their effects differ in intensity. Indeed NO is known to be an endogenous signalling molecule in the vascular system and besides its vasorelaxant property NO mediates several protective and beneficial functions of the endothelium, e.g. by inhibiting (1) neutrophil activation and adhesion, (2) platelet adhesion and aggregation, (3) vascular smooth muscle proliferation and (4) the expression of proinflammatory cytokines [22, 23]. Furthermore NO-mediated signalling is a component in other various physiologic processes, such as e.g. neurotransmission or immune defence. Nevertheless NO has also been implicated in the pathology of inflammatory diseases. Some of these processes are dependent on the recruitment of cGMP-mediated signalling, other are associated with direct interaction of NO or reactive nitrogen oxidative species derived from it with target proteins and do not involve the cGMP pathway [17]. Therefore further studies are required to clarify the underlying mode of action of the role of endogenous NO as wells as exogenous NO by means of NO donor administration on the induction of arteriogenesis.

The mode of administration of NO donors is important for another aspect. While the development of tolerances as well as the induction of endothelial dysfunction are well known disadvantages caused by the sustained, chronic exposure to organic nitrates, such as ISDN, NTG or isosorbide mononitrate (ISMN) [16, 24], the intermittent, transmucosal application of immediate-release NTG, i.e. sublingual NTG spray or tablet, does not bear any risk of these negative effects [25]–another advantage of the mode of administration in the present study setting. For the long-term administration of NTG formulations (e.g. by the use of transdermal NTG patches), oral slow-release ISDN formulations or long-acting ISMN a nitrate-free interval of 10–12 hours per day is generally recommended to prevent these adverse effects [16, 25].

Alongside an investigation of the pro-arteriogenic effect of intermittent short-acting NTG we were interested in a potential reversion of the anti-arteriogenic effect of ASA. Inhibition of collateral development by ASA has been previously shown in animal models of peripheral and cerebral vessel occlusion [26, 27]. The effect of administering ASA to the animals during the 5 or 10 days of the ROP appeared as expected to increase the severity of the infarct initiated by the FPO compared to the PBS and placebo controls; although, owing to the small sample size, there were few differences that reached statistical significance. Concomitant administration of NTG, however, appeared to counteract the ASA-induced inhibition of arteriogenesis to some extent. Although again not statistically different, the degree of ST-elevation and (at 10 days) the mean infarct size was lower for the animals that received ASA with NTG compared to those that received ASA with PBS or NTG-PLACEBO. Current guidelines for patients with coronary artery disease strongly recommend treatment with ASA [28, 29]. There is, therefore, a large population of patients that may benefit from NTG-induced collateral development or reversal of potential anti-arteriogenic triggers.

Whilst these data have demonstrated the arteriogenic potential of short-acting NTG, there are further clinical studies needed prior to this becoming a clinically accepted strategy for the treatment of occlusive vascular diseases. Dosages and dosing intervals would have to be carefully optimised in order to achieve the required response.

## Conclusion

Intermittent administration of short-acting NTG, encouraged the development of collateral blood vessels, reducing the severity of an induced myocardial infarction. Furthermore, the intermittent application of NTG appeared to be a stronger arteriogenic stimulus than the sustained bioavailability of slow-release ISDN. The short-acting NTG also attenuated the anti-arteriogenic activity of ASA. As a developed network of collateral vessels has the potential to reduce myocardial ischaemia caused by total coronary occlusion, these results suggest that, in addition to its use for alleviating acute angina, NTG therapy could play a further role in the treatment of patients with coronary artery disease or other occlusive vascular diseases, such as peripheral artery disease.
